# High expression of Claudin-2 in esophageal carcinoma and precancerous lesions is significantly associated with the bile salt receptors VDR and TGR5

**DOI:** 10.1186/s12876-017-0590-0

**Published:** 2017-02-17

**Authors:** Sohaib Abu-Farsakh, Tongtong Wu, Amy Lalonde, Jun Sun, Zhongren Zhou

**Affiliations:** 1 Department of Pathology and Laboratory Medicine, University of Rochester, Box 626, 601 Elmwood Ave, Rochester, NY 14642 USA; 2 Department of Biostatistics and Computational Biology, University of Rochester Medical Center, 265 Crittenden Boulevard CU 420630, Rochester, NY 14642-0630 USA; 3 Department of Medicine, Division of Gastroenterology and Hepatology, University of Illinois College of Medicine, 840 South Wood Street MC 716, Chicago, IL 60612 USA

**Keywords:** Claudin 2, Esophageal adenocarcinoma, Barrett’s esophagus, Tight junctions, VDR, TGR5

## Abstract

**Background:**

Claudins are a family of integral membrane proteins and are components of tight junctions (TJs). Many TJ proteins are known to tighten the cell structure and maintain a barrier. Claudin-2 forms gated paracellular channels and allows sodium ions and other small positively charged ions to cross between adjacent cells. Recently, we found that vitamin D receptor (VDR) enhanced Claudin-2 expression in colon and that bile salt receptors VDR and Takeda G-protein coupled receptor5 (TGR5) were highly expressed in esophageal adenocarcinoma (EAC) and precancerous lesions. Here, we examined the expression of Claudin-2 in EAC and precancerous lesions and its association with VDR and TGR5 expression.

**Methods:**

Claudin-2 expression was examined by immunohistochemistry on tissue microarrays, containing EAC, high grade dysplasia (HGD), low grade dysplasia (LGD), Barrett’s esophagus (BE), columnar cell metaplasia (CM), squamous cell carcinoma (SCC), and squamous epithelium (SE) cases. Intensity (0 to 3) and percentage were scored for each case. High expression was defined as 2–3 intensity in ≥ 10% of cells.

**Results:**

Claudin-2 was highly expressed in 77% EAC (86/111), 38% HGD (5/13), 61% LGD (17/28), 46% BE (18/39), 45% CM (29/65), 88% SCC (23/26), and 14% SE (11/76). It was significantly more highly-expressed in EAC, SCC and glandular lesions than in SE and more in EAC than in BE and CM. A significant association was found between Claudin-2 expression and VDR and TGR5 expression. No significant association was found between expression of Claudin-2 and age, gender, grade, stage, or patients’ survival time in EAC and SCC.

**Conclusions:**

We conclude that Claudin-2 expression is significantly associated with bile acid receptors VDR and TGR5 expression. Our studies identify a novel role of a tight junction protein in the development and progression of esophageal mucosal metaplasia, dysplasia and carcinoma.

## Background

The incidence of esophageal adenocarcinoma (EAC) has increased 700% in the United States over the past several decades [1]. Barrett’s esophagus (BE), an intestinal-like metaplasia of the distal esophageal mucosa, is a recognized precursor lesion and risk factor for EAC. Previous studies have suggested a sequence of events leading to EAC that starts from normal esophageal squamous epithelium to reflux esophagitis, followed by BE, dysplasia, and finally EAC [2]. The development and progression of these events is hastened by inflammation, bile salts, and acid reflux from gastro-esophageal reflux disease (GERD) [3–5].

Chronic exposure to bile salts in GERD promotes injury and inflammation of the esophageal epithelium and inhibits the Notch signaling pathway [6, 7]. Bile acids induce inflammatory gene expression and modulate inflammatory responses through the bile acid receptors including farnesoid X receptor (FXR), retinoic X receptor (RXR), TGR5 and Vitamin D receptor (VDR) [4, 8, 9]. They also alter gene expression by acting as ligands for nuclear receptors or by activating kinase signaling pathways [10, 11]. Bile acid receptors, including FXR, the Takeda G-protein-couples receptor 5 (TGR5) and VDR, have recently been identified in EAC and esophageal squamous cell carcinoma (ESCC) [4, 12–15]. We also showed that bile salts at pH of 5 destroyed intercellular junctions in squamous mucosa [16].

Claudins are a family of integral membrane proteins and are components of tight junctions (TJs) [17]. Many TJ proteins are known to tighten the cell structure and maintain a barrier [17, 18]. In contrast, Claudin-2 forms gated paracellular channels and allows sodium ions and other small positively charged ions to cross between adjacent cells [19–22]. Claudin-2 expression may be involved at early stages of transformation in inflammatory bowel disease-associated neoplasia [23]. Claudin-2 was found in various human cancers including breast, ovarian, urothelial, colorectal, prostate, and gastric cancers linking to better or worse prognosis [24–28]. Recently, we identified Claudin-2 as a target gene of VDR in colonic epithelial cells [29]. Our study has demonstrated that bile salt receptors VDR and TGR5 were highly expressed in EAC and precancerous lesions [29, 30]. However, the relationship between Claudin-2 and bile salt receptors in EAC and esophageal precancerous lesions is still unknown.

In the current study, we used immunohistochemical methods to investigate the expression of the tight junction protein Claudin-2 in EAC, esophageal precancerous lesions, and esophageal squamous cell carcinoma. The association of Claudin-2 with bile salt receptors VDR and TGR5 was also investigated.

## Methods

### Patients for tissue microarrays

All 111 patients with EAC used for tissue microarrays (TMAs) construction were treated with esophagectomy at the Strong Memorial Hospital/University of Rochester between 1997 and 2005 (99 male [89%], 12 female [11%]). The patient age ranged from 34 to 85 years with a mean of 64 years. The follow-up period after esophagectomy ranged from 0.3 to 142 months with a mean of 39 months.

### Construction of tissue microarray

TMAs containing material from 39 cases of BE, 65 cases of columnar cell metaplasia (CM), 76 cases of squamous epithelium (SE), 28 cases of low grade dysplasia (LGD), 13 cases of high grade dysplasia (HGD), 111 cases of esophageal adenocarcinoma (EAC), and 26 cases of esophageal squamous cell carcinoma (ESCC) were constructed from representative areas of formalin-fixed specimens collected during 1997 through 2005 at the Department of Pathology and Laboratory Medicine, University of Rochester Medical Center, Rochester, NY. Five-micron sections were cut from TMAs and stained with hematoxylin and eosin to confirm the presence of the expected tissue within each tissue core. Additional sections were cut for IHC staining. Some tissue cores in TMAs were falloff from slides during processing and were excluded from our study. The research project was approved by Research Subjects Review Board committee in University of Rochester (RSRB00028546).

### Immunohistochemical staining

Tissue sections from the TMA were deparaffinized, rehydrated through graded alcohols, and washed with phosphate-buffered saline. Antigen retrieval was performed by heating sections in 10 mM citrate (pH 6.0) boiling buffer for 15 min. The tissues were permeabilized with 0.3% Triton X for 1 h at room temperature. After endogenous peroxidase activity was quenched and nonspecific binding was blocked, mouse monoclonal anti-Claudin-2 (1:200; Santa Cruz Biotechnology, Santa Cruz, CA), anti-VDR (1:100; Santa Cruz Biotechnology, Santa Cruz, CA) and anti-TGR5 antibodies (1:200; Santa Cruz Biotechnology, Santa Cruz, CA) was incubated at 4 °C overnight. Biotinylated secondary antibody (Jackson ImmunoResearch Laboratories, West Grove, PA) was allowed to incubate for 1 h. After washing, sections were incubated with avidin-biotin–peroxidase complex (Vector Laboratories, Burlingame, CA) for 1 h at room temperature. For color reaction development, slides were immersed in Vector NovaRed substrate (Vector Laboratories, Burlingame, CA) for 2 min and counterstained with Flex Hematoxylin for 2 min (Vector Laboratories, Burlingame, CA). A negative control was performed by replacing anti-VDR antibody with normal serum.

### Scoring of IHC staining

**Fig. 1:**
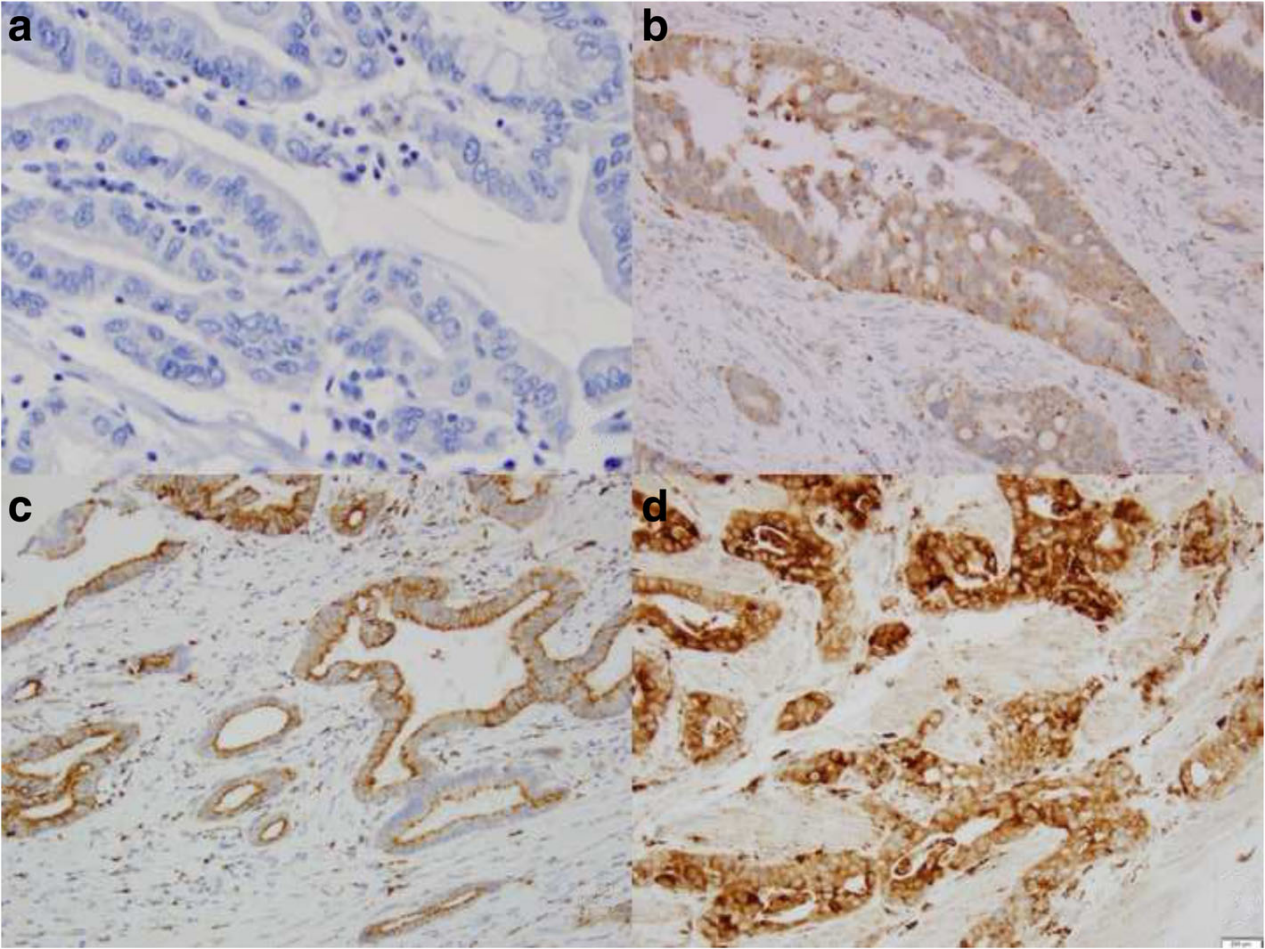
Immunostain score of Claudin-2 expression in esophageal adenocarcinoma. **a** No staining (0); **b** 1+ staining, **c** 2+ staining, and **d** 3+ staining

All sections were reviewed independently by Z.Z. and S.A., who were blinded to all clinical and pathologic information. Discordant cases were reviewed by both investigators, and a consensus was reached. For Claudin-2 IHC stain, the percentage of positive cells was determined. The intensity of staining was graded 0, 1+, 2+, or 3+. Claudin-2 was considered to be highly expressed if 10% or more of the cells stained with an intensity score of 2+ or 3+ (Fig. 1).

### Statistical analysis

All statistical tests were 2-sided. *P <*0 .05 was considered to be statistically significant. Kaplan-Meier survival estimator with log-rank test was used to analyze the patient survival rates in the Claudin-2 high expression group versus the non-high expression group. The χ2 or Fisher exact tests were used to compare Claudin-2 positivity rates between EAC, HGD and LGD, BE, non–goblet cell metaplasia, and SE subcategories as appropriate. Statistical analyses were performed using SAS version 9.3 (SAS, Cary, NC).

## Results

### High expression of Claudin-2 in precancerous lesions, EAC, and ESCC

**Fig. 2:**
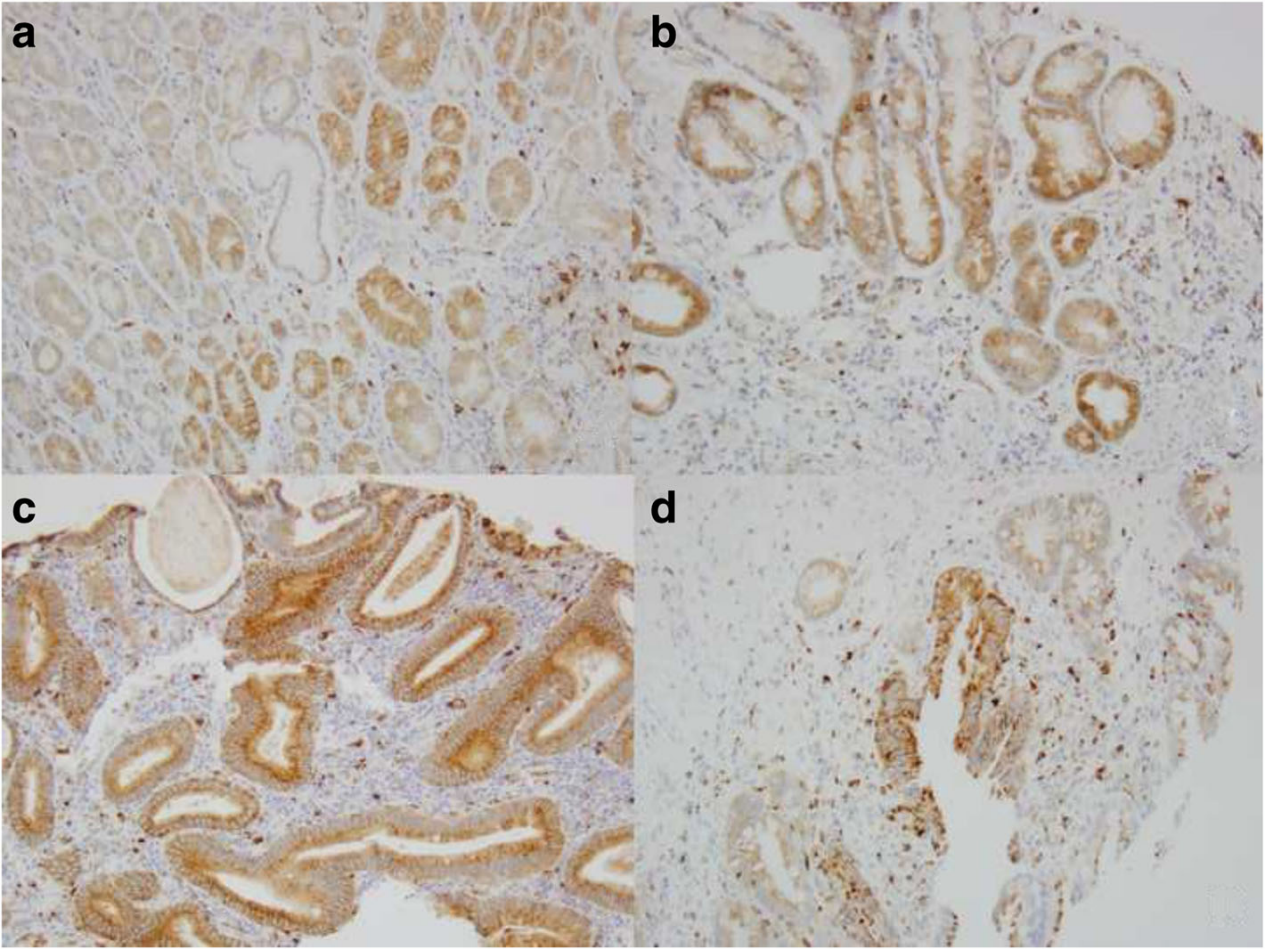
Claudin-2 expression in esophageal precancerous lesions. **a** Cardiac mucosa with 2+ immunostaining; **b** Barrett’s esophagus with 3+ immunostaining; **c** low grade dysplasia with diffuse 3+ immunostaining; **d** high grade dysplasia with focal 2+ immunostaining

**Fig. 3:**
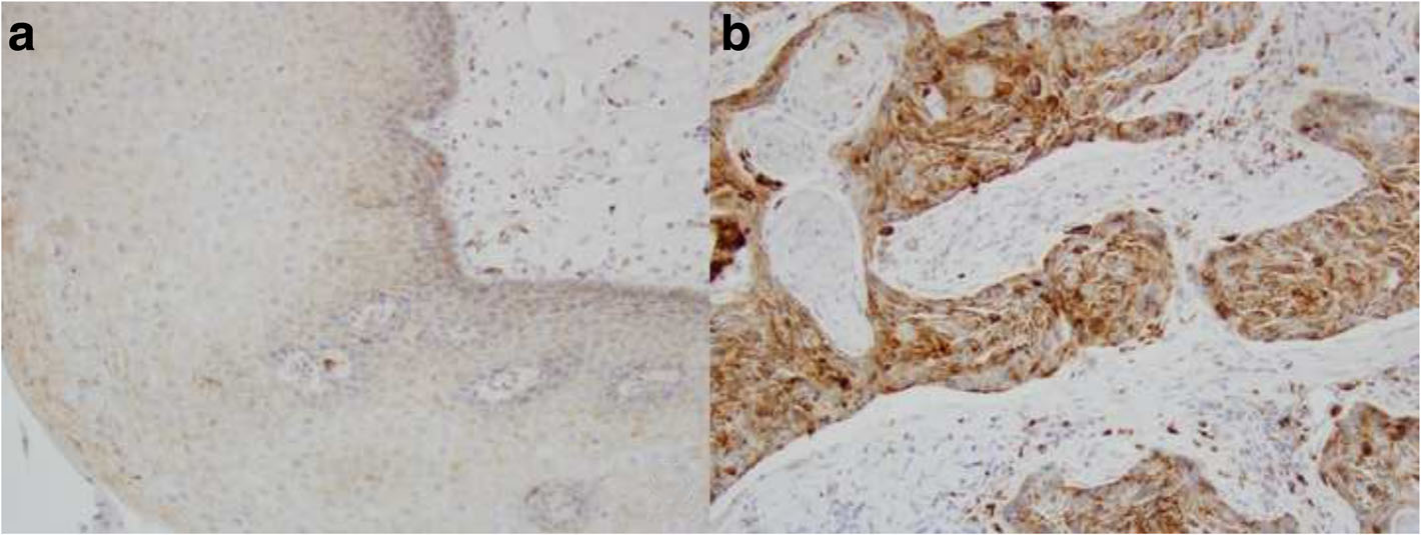
Comparison of Claudin-2 expression between normal squamous epithelium and squamous cell carcinoma. **a** in normal squamous epithelium with the immunostain score is 1+; **b** in squamous cell carcinoma with the immunostain score is 3+

Claudin-2 immunostaining is located at cytoplasm and membrane, but predominantly at the cell and the basal membrane of the glands and squamous mucosa. It diffusely distributes in most of glands in columnar cell metaplasia, BE, dysplasia and EAC (Fig. 1 and Fig. 2). Claudin-2 was highly expressed in 77% EAC (86/111), 38% HGD (5/13), 61% LGD (17/28), 46% BE (18/39), 45% CM (29/65), 88% SCC (23/26), and 14% SE (11/76) (see Table 1). It is significantly more expressed in EAC than in HGD (*p =* 0.0055), BE (*p =* 0.0004) and CM (*p <* 0.0001), and significantly more expressed in both BE and CM than in SE (*p =* 0.0004 and 0.0001 respectively). It is also more expressed in SCC than in SE (*p <* 0.0001) (Fig. 3). No significant difference was found between the levels of Claudin-2 expression in CM, BE, LGD, and HGD.

**Table 1:** Rate of Claudin-2 high expression in EAC and precancerous lesions and squamous cell carcinoma

Histological Type	Total (n)	High-expression (%)	Non-high expression (%)
Adenocarcinoma	111	86 (77%)	25 (23%)
High grade dysplasia	13	5 (38%)	8 (62%)
Low grade dysplasia	28	17 (61%)	11 (39%)
Barrett’s esophagus	39	18 (46%)	21 (54%)
Columnar cell metaplasia	65	29 (45%)	36 (55%)
Squamous epithelium	76	11 (14%)	65 (86%)
Squamous cell carcinoma	26	23 (88%)	3 (12%)

### Survival rate analysis in EAC cases

**Fig. 4:**
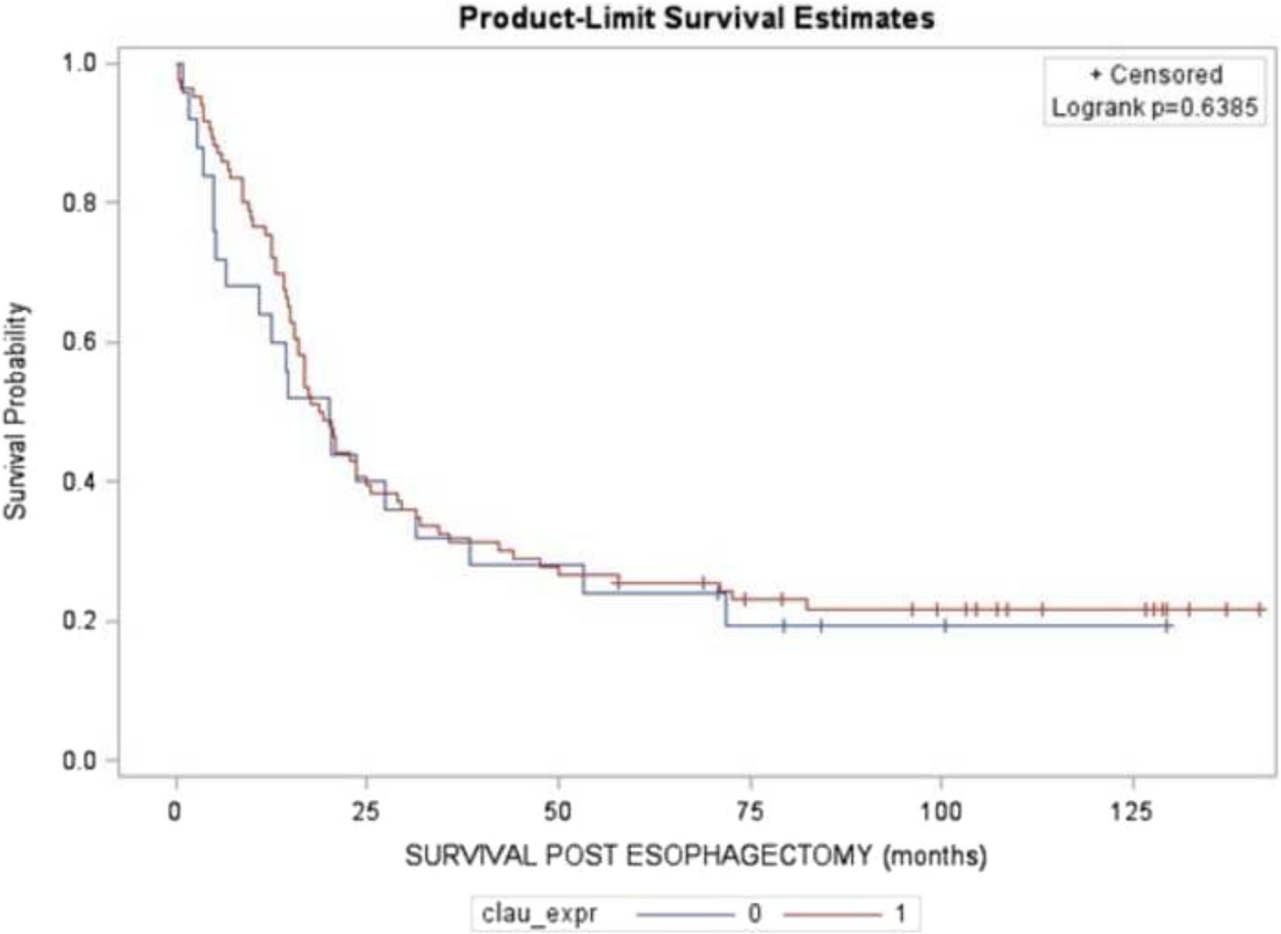
Kaplan-Meier analysis of overall survival associated with Claudin 2 high expression and non-high expression in esophageal adenocarcinoma

Kaplan-Meier analysis was used to calculate the survival curves of Claudin-2 high and non-high expression groups. Log-rank test was used to compare the effect of Claudin-2 expression in survival rates for patients with esophageal adenocarcinoma (Fig. 4). The median survival time in the Claudin-2 high expression group by immunostain was 19 months with a mean survival time of 40 months. The Claudin-2 non-high expression group had a median survival time of 20 months with a mean survival time of 33 months (censoring rate = 22%). The log-rank test failed to reveal significant differences in the survival time for the Claudin-2 high expression and non-high expression group (*p =* 0.6385; Fig. 4).

### Association of high Claudin-2 expression with clinicopathologic characteristics of EAC

The association of Claudin 2 high expression with clinicopathologic features in esophageal adenocarcinoma was analyzed. None of the clinicopathologic characteristics including age, sex, TNM staging and differentiation were found to be significantly associated with Claudin-2 high expression (Table 2).

**Table 2:** Examination of relationship of Claudin-2 high expression and clinicopathologic characteristics in esophageal adenocarcinoma

Covariate		High-expression	Non-high expression	*P* value
Age	Mean (SD)	63.9 (11.1)	63.6 (11.4)	0.9128
	Range	34 – 84	40 – 85	
Gender	Male	75	24	0.2922
	Female	11	1	
Lymph node metastasis	# (+) nodes	4.2 (5.2)	3.7 (4.5)	0.6286
Survival time		39.51 (41.55)	33.32 (35.88)	0.6385
Tumor location *Fisher’s Test*	DISTAL	19	4	0.7162
	GEJ	64	21	
	Other	3	0	
Tumor location *Fisher’s Test*	DISTAL	19	4	0.8078
	GEJ	64	21	
	MID	2	0	
	PROXIMAL	1	0	
TNM Stage *Fisher’s Test*	1	1	0	0.6444
	2	10	1	
	3	21	8	
	4	54	16	
T stage *Fisher’s Test*	1	2	0	0.4647
	2	13	1	
	3	17	6	
	4	54	18	
N stage *Fisher’s Test*	0	20	6	0.7982
	1	42	13	
	2	14	5	
	3	10	1	
Differentiation (missing = 2) *Fisher’s Test*	Poor	57	15	0.7075
	Moderate	23	9	
	Well	4	1	

### Association of high Claudin-2 expression with high TGR5 and VDR expression

**Fig. 5:**
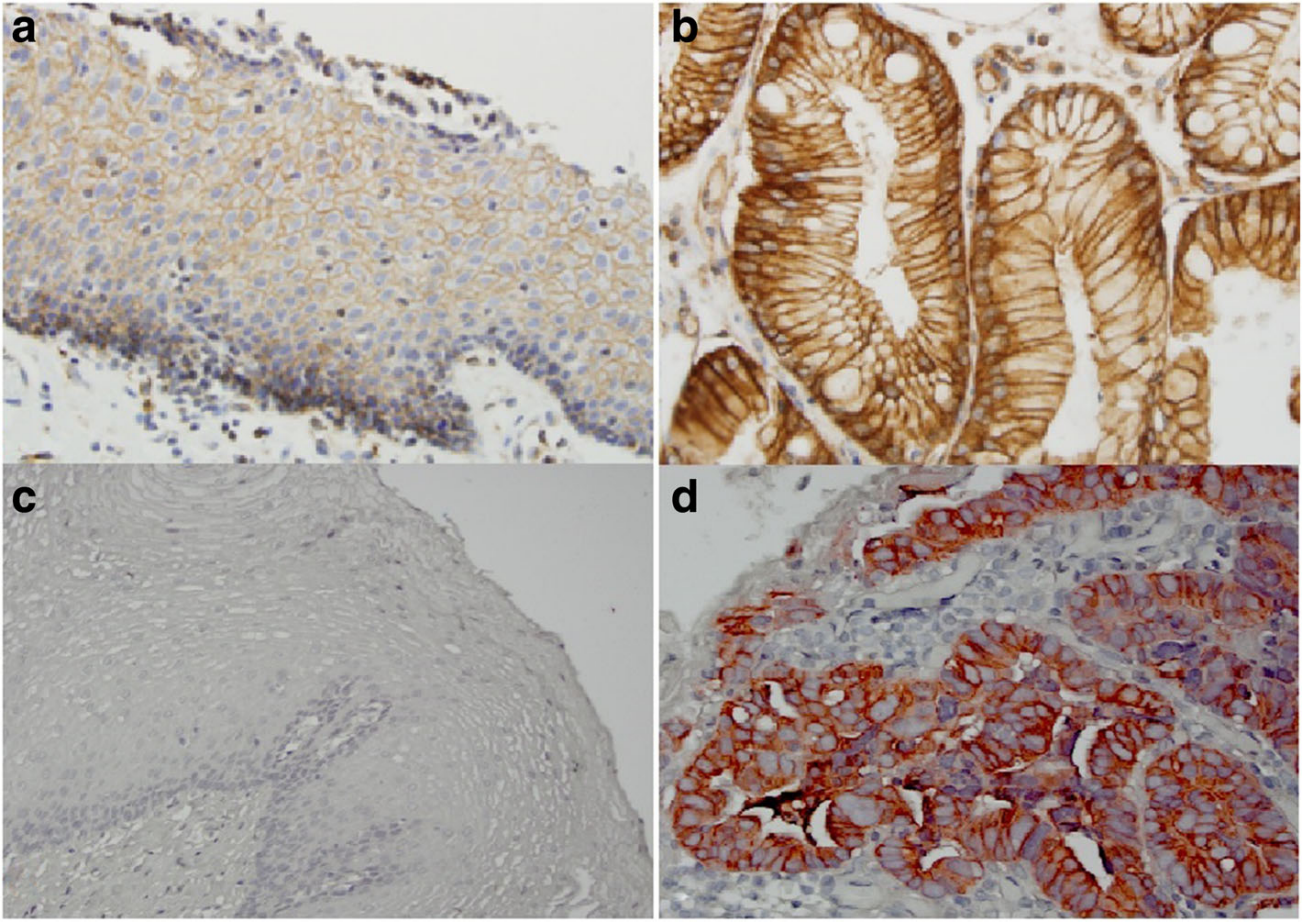
TGR5 and VDR expression in esophagus. **a** TGR5 is moderately expressed in whole squamous mucosa. Focal extensive TGR5 expression is present in the basal membrane. **b** TGR5 expression is located at cell membrane and distributes the whole glands in BE case. **c** VDR is not expressed in squamous mucosa. **d** VDR expression is located at cell membrane and cytoplasm, predominately in cell membrane and distributes the whole glands in HGD case

VDR expression is located at both cytoplasm and cell membrane, but TGR5 predominately at cell membrane (Fig. 5) [14, 31]. TGR5 is low or moderately positive on whole layer of squamous mucosa (Fig. 5a), but VDR usually is not present on squamous mucosa and ESCC (Fig. 5c). VDR and TGR5 expression diffusely distribute in columnar cell metaplasia, dysplasia and EAC, which is similar to the distribution of Claudin-2 (Fig. 5c and d). We further compared the expression level of Claudin-2 with TGR5 and VDR in all cases and then separately for EAC. The positive correlations of Claudin-2 high expression with TGR5 and VDR were statistically significant for the full samples (*p =* 0.0051 and 0.0046, respectively, Table 3), but Claudin-2 is not significantly associated TGR5 and VDR in EAC cases only (*p = 0.86 and 0.65*).

**Table 3:** Association between Claudin-2 high expression and each of TGR5 and VDR high expression across all cases

	Claudin-2 High-expression	Claudin-2 Non-high expression	*P* value
TGR5 High-expression	113	76	0.0051*
TGR5 Non-high expression	76	93	
VDR High-expression	117	83	0.0046*
VDR Non-high expression	52	71	

## Discussion

In the current study, we show that tight junction protein Claudin-2 is localized to the cytoplasm and cell membrane of squamous cell and glandular cells. The proportion of cases with high Claudin-2 expression showed an upward trend from squamous mucosa to precancerous lesions to EAC. Claudin-2 was also highly expressed in esophageal squamous cell carcinoma. Claudin-2 expression positively correlated with the expression of the bile acid receptors VDR and TGR5 in esophageal tissue.

Bile acid reflux, in addition to acidic pH, is required to cause dilation of intercellular spaces in esophageal epithelium in vitro, as we showed in a previous study [16]. Another study using rat model with esophagojejunostomy and gastrectomy demonstrated that bile acids but not gastric acids induced the transition to BE [32]. We recently found that bile salt receptors VDR and TGR5 were highly expressed in esophageal adenocarcinoma (EAC) and precancerous lesions [14, 30]. The above studies might suggest that bile acids through VDR and TGR5 receptors play an important role in the dilation of intercellular spaces and in the development of Barrett’s esophagus.

VDR was also found to directly enhance Claudin-2 expression in intestinal epithelium [29, 33]. In addition, deoxycholic acid (DCA) and trypsin in the higher concentration of 2.5 mM can decreased the resistance of GERD patients’ squamous mucosa and the claudin-3, −4 and E-cadherin expressions [18]. However, the Claudin-2 expression is found at basal and suprabasal zone of the squamous mucosa, but did not change significantly in GERD patients. We found that Claudin-2 has similar distribution in squamous mucosa compared to TGR5 and similar distribution in glandular cells compared to both TGR5 and VDR. In addition, Claudin-2 expression was positively correlates with the VDR and TGR5 expression. These data support our hypothesis the bile acids induce Claudin-2 expression through VDR and TGR5. Claudin-2 is a unique protein in the Claudin family and forms a cation and water selective paracellular channel in tight junctions [19, 34, 35], and its expression increases intercellular permeability which opens the gate to change the microenvironment of the esophageal epithelium and may eventually lead to columnar cell metaplasia and BE.

Based on the in vitro experiments and animal models discussed earlier, potential bile acid blocking drugs in the future might be able to reduce the expression of Claudin-2 and decrease the risk of progression to BE. However, our study utilizes an immunohistochemical method to detect the expression of Claudin-2; it has the limitation to directly prove the functional relationship between Claudin-2 and bile acid receptors. This functional relationship will be studied in future.

The rate of high expression of Claudin-2 was significantly increased from 14% in SE to 45% in columnar cell metaplasia and BE. This is consistent with the results of a previous study that found Claudin-2 overexpression in BE [36]. Mullin et al. found that leak of sucrose in the urine dramatically increased about 2 folds in esophagitis and 3 folds in BE, and that Claudin-2 expression increased 225 folds since the normal squamous epithelium showed almost no expression of Claudin-2. Some studies also showed that Claudin-1, −2, and −4 were significantly changed in GERD patients both at the transcript and protein levels compared to normal patients [18, 37]. Weimann et al. compared six immunohistochemical markers for the histologic diagnosis of neoplasia in Barrett’s esophagus [38]; however, they found that Claudin-2 staining was only focal and weak and did differ significantly between normal (5%), Barrett’s esophagus (2%), low- (5%) and high-grade dysplasia (7%) and EAC (16%). Our study showed that it was significantly more expressed in EAC than in HGD (*p =* 0.0055), BE (*p =* 0.0004) and CM (*p <* 0.0001), and significantly more expressed in both BE and CM than in SE (*p =* 0.0004 and 0.0001 respectively). The reason for the discordant results between their study and ours is not completely clear; however, we suggest that the antibodies used might be a possible reason. They used an anti-Claudin-2 rabbit polyclonal antibody (Panomics, Redwood City, CA, USA) and we used an anti-Claudin-2 mouse monoclonal antibody (Santa Cruz, CA, USA). In addition, our antibodies were validated by Western Blot in a previous study [29]. Furthermore, the number of the cases in each study was different; they had a relatively small number of samples in each group.

Studies have shown that different Claudins can be over or under-expressed in various human cancers including breast, ovarian, urothelial, colorectal, prostate, and gastric cancers. Their over or under-expression has been linked to better or worse prognosis in some cancer types [24–27]. In the esophagus, Claudins-3, −4 and −7 were reported to have increased expression in esophageal adenocarcinoma [39]. In our study, we found that Claudin-2 was more highly expressed in EAC compared to precancerous lesions and normal esophageal squamous mucosa, suggesting that Claudin-2 might have a role in the development and progression of EAC. However, we did not find a significant correlation between Claudin-2 expression in EAC and patient’s survival or other clinicopathologic features. Claudin-2 was also overexpressed in ESCC; no correlation was identified between Claudin-2 expression in ESCC and patient’s survival.

## Conclusion

In summary, we conclude that Claudin-2 expression is significantly increased from normal squamous mucosa to columnar cell metaplasia, BE, low- and high-grade dysplasia to EAC. The expression of Claudin-2 positively correlates with the expression of the bile acid receptors VDR and TGR5. This implies that bile acid reflux may induce Claudin-2 over expression and increase the risk of the development of BE. Our study provides new insights into the role of a tight junction protein and bile acid receptors in the pathogenesis of Barrett’s esophagus and esophageal cancer.

## Abbreviation

BE: Barrett’s esophagus
CM: Columnar cell metaplasia
EAC: Esophageal adenocarcinoma
GERD: Gastroesophageal reflux disease
HGD: High grade dysplasia
IHC: Immunohistochemistry
LGD: Low grade dysplasia
SCC: Squamous cell carcinoma
SE: and squamous epithelium
TGR5: The G-protein couples bile acid receptor
TMA: Tissue microarray
TNM: Tumor node metastasis
VDR: Vitamin D receptor
